# Pathogenicity and virulence of human respiratory syncytial virus: Multifunctional nonstructural proteins NS1 and NS2

**DOI:** 10.1080/21505594.2023.2283897

**Published:** 2025-09-16

**Authors:** Trudy N. Merritt, Jingjing Pei, Daisy W. Leung

**Affiliations:** Department of Medicine, Washington University School of Medicine, St. Louis, MO, USA

**Keywords:** Human respiratory syncytial virus, nonstructural proteins, NS1, NS2, viral IFN antagonists

## Abstract

Human respiratory syncytial virus (hRSV) is a major cause of lower respiratory tract infections in young children, the elderly, and immunocompromised. Despite its discovery over 60 years ago and the global impact on human health, limited effective prophylactic or therapeutic options have been available for hRSV infections. This has largely been attributed to the legacy of vaccine failure in the 1960s using a formalin-inactivated RSV , which led to enhancement of disease post exposure to hRSV infection and hampered subsequent development of vaccine candidates. Recent FDA approval of vaccines for older adults and pregnant individuals are major advancements but leaves children between 6 months and 5 years of age unprotected. Part of this limitation is due to a lack of complete understanding of the factors that contribute to hRSV pathogenesis. The nonstructural proteins NS1 and NS2 are multifunctional virulence factors unique to hRSV and that play critical roles during hRSV infection, including antagonizing interferon (IFN) signaling to modulate host responses to hRSV infection. However, the molecular mechanisms by which the nonstructural proteins mediate their IFN inhibitory functions have not been completely defined. Current progress on the characterization of NS1 and NS2 during infection provides deeper insight into their roles. Furthermore, reverse genetics systems for hRSV provide a viable strategy to generate attenuated viruses by introduction of select mutations while maintaining immunogenicity required to elicit a long-term protective response. Here, we will review the current state of knowledge of the nonstructural proteins, their contributions to RSV pathogenesis, and their potential as targets for therapeutic development.

## hRSV disease

Human respiratory syncytial virus (hRSV) is a human pathogen that circulates seasonally as many other respiratory viruses. hRSV spreads readily through respiratory droplets and can cause mild symptoms in an average adult similar to the common cold, such as runny nose, coughing, and fever. In some cases, hRSV infection in the upper respiratory tract can lead to spread and more severe symptoms in the lower respiratory tract, especially in children under the age of 2 and adults older than 65 years of age. Severe symptoms include wheezing, difficulty breathing within 3–7 days after initial exposure, bronchiolitis, inflammation, and pneumonia [1–3]. Severity of disease is compounded by comorbidities such as chronic lung or heart disease and weakened immune systems. In the United States, hRSV poses a significant public health burden with socioeconomic impact. The Centers for Disease Control and Prevention (CDC) estimates that hRSV causes hospitalizations of over 60,000 children under the age of 5 and 180,000 adults over the age of 65 and deaths up to 500 children and 14,000 older adults [4–12]. These numbers are likely greater when considering the number of undiagnosed or unreported cases as well as global hospitalization and mortality rates for hRSV infections [13,14].

## Viral genome

**Figure 1.**
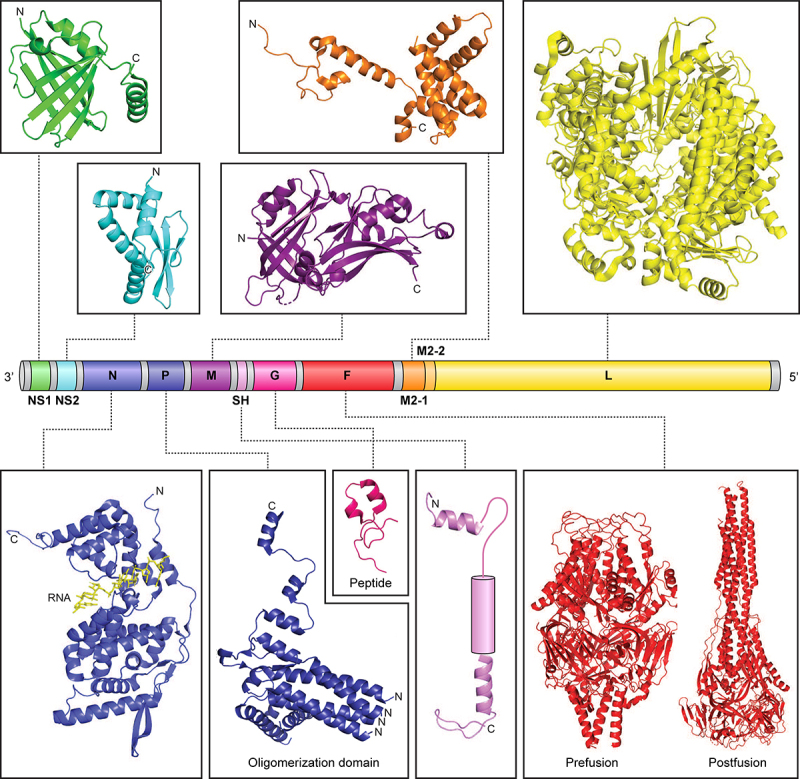
hRSV genome and viral proteins. Domain organization of the hRSV RNA genome (A2 strain). The viral genome contains 10 genes encoding for 11 proteins. The M2 gene contains two ORFs encoding for M2–1 and M2–2 proteins. Shown above and below the genome are representative structures available for the different hRSV proteins (NS1, PDB 5VJ2; NS2, PDB 7LDK; N, PDB 2WJ8; P, PDB 6PZK; M, PDB 2QP; SH, PDB 2NB7 and 2NB8; G, PDB 6BLH; F, PDB 4JHW and 3RRR; M2–1, PDB 4C3D; and L, 6PZK). For SH, NMR coordinates are not available for the transmembrane helix and is represented as a rounded cylinder. NS1, nonstructural protein 1; NS2, nonstructural protein 2; N, nucleoprotein; P, phosphoprotein; M, matrix; SH, small hydrophobic; G, glycoprotein; F, fusion glycoprotein; M2–1, transcription processivity factor; M2–2, replication co-factor; L, large polymerase.

hRSV is a pleiomorphic enveloped virus, with spherical and filamentous forms [15–18], that belongs to the *Pneumoviridae* family of nonsegmented, negative sense RNA viruses (NNSVs). hRSV along with bovine respiratory syncytial virus (bRSV) and murine pneumonia virus (MPV) (formerly pneumonia virus of mice or PVM) are in the *Orthopneumovirus* genus, whereas human metapneumovirus (HMPV) is in the *Metapneumovirus* genus [15,19–21]. hRSV is further classified into two subtypes A and B (A1, A2, B1, and B2). The prototypical genome of hRSV is approximately 15.2 kilobases (strain A2) encoding for 10 genes and 11 open reading frames (ORFs) in the order of 3’-NS1-NS2-N-P-M-SH-G-F-M2-L-5’ (Figure 1). Like other NNSVs, replication of the negative-sense genome in cytoplasmic inclusion bodies requires generation of a positive-sense antigenome that serves as a template for RNA synthesis [22,23]. Nucleoprotein (N) encapsidates the viral RNA genome and is part of the viral replication complex that is bridged by phosphoprotein (P) to the large RNA-dependent RNA polymerase (L) [24–27]. L contains domains required for RNA synthesis including an RdRp domain, an mRNA capping domain, and a methyltransferase domain. Recent structures of hRSV L bound to tetrameric P revealed that different regions in L interact with a conformationally dynamic P (Figure 1).

**Figure 2.**
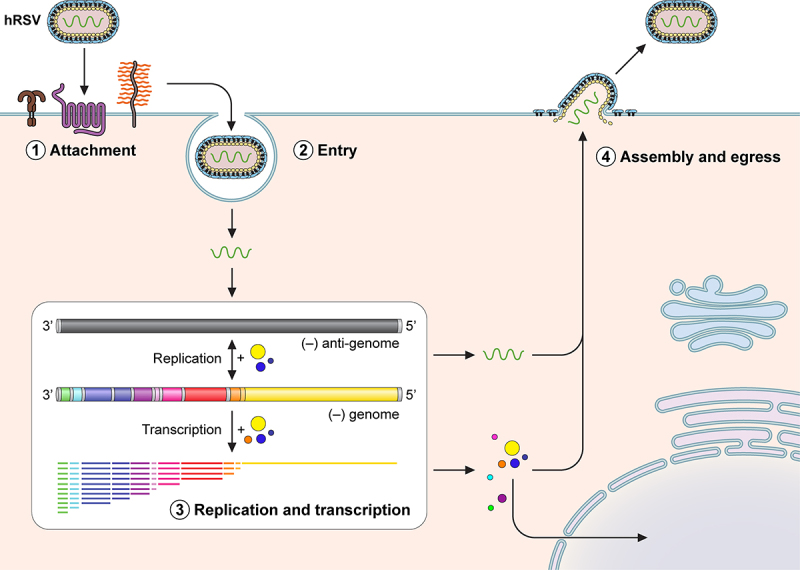
hRSV replication cycle. Simplified schematic of the different stages of the viral replication cycle. 1, Several host factors are involved in attachment of hRSV to the host membrane that is cell type dependent. 2, Entry may involve endocytosis or micropinocytosis of the virion followed by fusion of viral and host cell membranes. 3, Replication and transcription occurs in inclusion bodies where the negative strand genome serves as template for generating the antigenome. The negative strand genome is also transcribed by a sequential start-stop mechanism that results in a gradient of mRNAs from the 3’ to the 5’ end. 4, After viral proteins are generated, some like NS1 and M partially localize to the nucleus and are later assembled along with the viral genome to produce new infectious viral particles.

Transcription proceeds by a sequential start-stop mechanism that results in a gradient of viral mRNA abundance from the 3’ end to the 5’ end (Figure 2) [28]. The switch from replication to transcription involves cofactor M2–1, which binds to N as well as RNA and functions as an anti-terminator and processivity factor [29–32]; M2–2 likely is involved in the switch from transcription to replication [33]. The first two genes that are proximal to the promoter and transcribed in great abundance are nonstructural 1 and 2, which encode for the NS1 and NS2 proteins that function as interferon (IFN) antagonists. This is followed by N, P, matrix (M), small hydrophobic (SH), glycoprotein (G), fusion (F), M2, and L. M is required for assembly along with G, F, and SH, which are surface transmembrane glycoproteins that function in attachment and entry [34,35].

## Viral entry

hRSV infects ciliated epithelial cells in the upper respiratory tract and can spread to the bronchiolar epithelium and alveolar cells in the lower respiratory system, including type I pneumocytes [36,37]. Viral entry involves attachment to the cell membrane and fusion of the viral and cell membranes. Attachment to the cell is largely mediated by the G glycoprotein, which is genetically diverse between hRSV A and B subtypes. G is highly glycosylated and attaches to heparan sulphate proteoglycans (HSPGs) that are present on immortalized cells [38,39]. However, HSPGs are absent in pulmonary cells that are infected by hRSV. Subsequent studies identified that CX3C chemokine receptor (CX3CR1), which is expressed in ciliated lung epithelial cells, is important for interactions with hRSV G and for viral attachment [40]. F glycoprotein, the sequence of which is highly conserved compared to G, is also involved in attachment and can interact with nucleolin [41], epidermal growth factor receptor (EGFR) [42], insulin-like growth factor-1 receptor (IGF1R) [43], and intercellular adhesion molecule-1 (ICAM-1) [44]. However, hRSV F primarily facilitates fusion of viral and host membranes through interactions with other host receptors. After F is processed and trimerized, F undergoes conformational changes from a prefusion form to a postfusion form that allows insertion of F into the host membrane (Figure 1). SH forms an ion channel and may be necessary to inhibit TNF-α mediated apoptosis; however, the importance of hRSV SH is less clear as SH is not required to form an infectious virion [45–48]. The presence of host receptors vary by cell type and elucidating the mechanism of host cell attachment by RSV F and G remains an open topic in the field that may involve a two-step fusion event or micropinocytosis followed by endosomal fusion and is reviewed elsewhere [49].

## IFN signaling in response to viral infection

**Figure 3.**
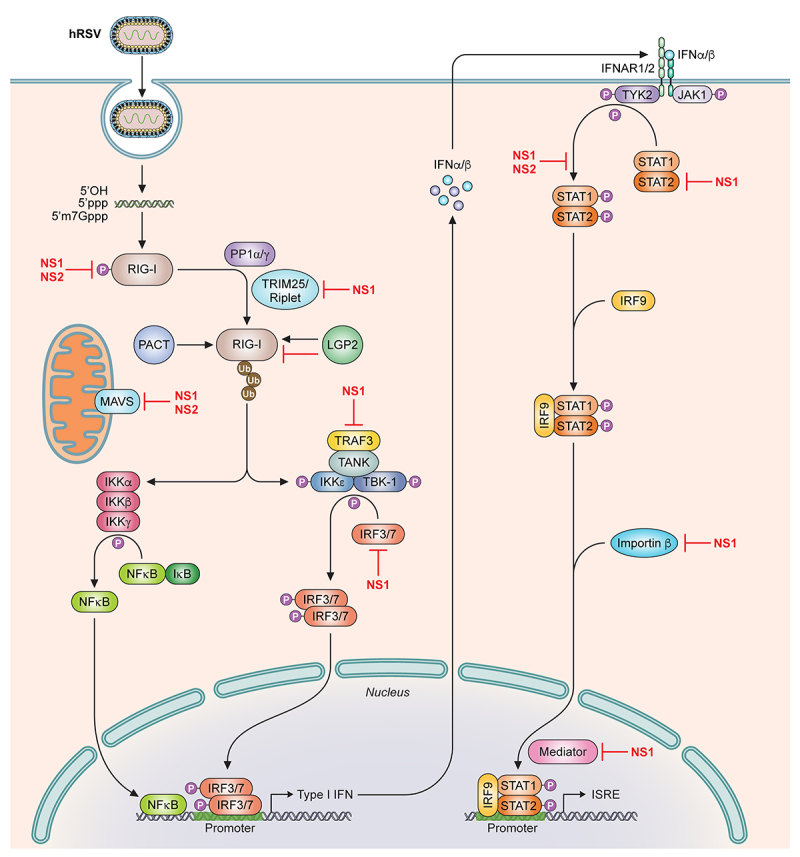
NS1 and NS2 antagonize multiple targets in the IFNα/β signaling pathway. Simplified representation of the IFNα/β induction and response signaling pathway. Cytosolic pattern recognition receptors, including RIG-I and MDA5, detect viral pathogen associated molecular patterns and stimulate the induction of IFNα/β. IFNα/β stimulates other downstream signaling pathways to activate the transcription of IFN-stimulated genes (ISG). hRSV NS1 and NS2 inhibit the function of different molecules at points indicated on the pathway. 5’OH, 5’-hydroxyl; 5’ppp, 5’-triphosphate; 5’m7Gppp, 5’-7-methylguanosine cap; PP1α/γ, protein phosphatase 1α/γ; RIG-I, retinoic inducible gene-I; MDA5, melanoma differentiation-associated protein 5; TRIM25, tripartite motif-containing protein 25; PACT, protein activator of PKR; LGP2, laboratory of genetics and physiology 2; MAVS, mitochondrial antiviral-signaling protein; NF?B, nuclear factor kappa-light-chain-enhancer of activated B cells; TRAF3, TNF receptor associated factor 3; TANK, TRAF family member-associated NF?B activator; I?B, inhibitor of NF?B; IKK, I?B kinase; TBK-1, TANK-binding kinase 1; IRF, interferon regulatory factor; IFN, interferon; IFNAR1/2, IFN alpha and beta receptor subunit 1/2; JAK1, Janus kinase1; Tyk2, non-receptor tyrosine kinase 2; STAT, signal transducer and activator or transcription; ISRE, IFN stimulated response elements; Ub, ubiquitin; P, phosphorylated.

Type I IFN signaling stimulates host innate immune responses and is a self-defence mechanism to combat viral infection and to prevent viral spread [50,51]. The RIG-I like receptors (RLR) RIG-I and MDA5 are cytosolic pattern recognition receptors (PRR) that sense pathogen associated molecular patterns (PAMPS), which include viral RNAs [52–59]. Activation of RLRs results in the association of several adapter molecules, such as MAVS (also referred to IPS-1/Cardif/Visa), TRAF, and TRIF, that then recruits downstream signaling kinases TBK1, IKK? and NK?B (Figure 3). These activated kinases can subsequently phosphorylate the transcription factors IRF3 and IRF7, which dimerize and translocate into the nucleus to induce Type I IFN production, mainly IFNα/β. IFNα/β can then act in an autocrine or paracrine manner and signal through binding to the Type I IFN receptor (IFNAR1/2) leading to stimulation of the JAK-STAT signaling pathway. This activates the critical transcription factors STAT1 and STAT2, which homodimerizes or heterodimerizes and binds to IRF9, and localizes in the nucleus to induce transcription of IFN-stimulated response elements (ISRE), or antiviral genes, to establish an antiviral state [60–62].

## hRSV encoded IFN antagonists

Early studies of the hRSV nonstructural proteins revealed that NS1 and NS2 function as IFN antagonists, especially during the early stages of infection given their proximity to the 3’ UTR [63,64]. NS1 and NS2 are found only in RSV (human, bovine, ovine, caprine, pneumovirus of mouse) and not in any of the other *Mononegavirales* viruses, including the closely related HMPV. The viral genome of the related paramyxoviruses or filoviruses lacks the NS1 and NS2 counterparts but encodes for accessory proteins, such as the V, W, and C proteins in paramyxoviruses or VP24 and VP35 in filoviruses, that have critical roles in antagonizing interferon (IFN) responses [65,66]. Comparison of the NS1 and NS2 sequences reveals little sequence similarity to these accessory proteins and approximately 12% identity between NS1 and NS2, most notably in the last four residues encoding Asp-Leu-Asn-Pro. The functional significance of this motif is not clear, although there may be a role for these residues in mediating protein–protein interactions [67].

Recombinant human RSVs (hRSVs) where deletion of NS1, NS2, or both genes (NS1, NS2, NS1/NS2) significantly increased IFNβ mRNA levels compared to wildtype hRSV infected cells, suggesting that both NS1 and NS2 suppressed IFN signaling [68–72], similar to that observed for bovine NS1 and NS2 [73,74]. NS1 and NS2 encode for proteins with approximate molecular weights of 15 and 14 kDa, respectively [75–77], but are not considered to be part of the mature virion [78], hence their designation as nonstructural proteins. It is not fully understood why RSV requires two proteins to mediate this specific function. However, recent studies provide evidence that NS1 and NS2 are multifunctional proteins that likely target different molecules in the IFN signaling pathway to suppress IFN production and responses cooperatively and potently during hRSV infection to facilitate viral pathogenesis (Figure 3).

## Cytosolic NS1-mediated inhibition of the IFN signaling pathway

Given that stimulation of IFN production depends on PRR activity, it is not surprising then that hRSV targets this crucial first step in the pathway, likely using multiple mechanisms. RIG-I is a cytoplasmic PRR that exists in a phosphorylated and autoinhibited conformation. Binding of viral RNA PAMPS to its C-terminal domain relieves autoinhibition and allows for the dephosphorylation and ubiquitination of the tandem N-terminal caspase activation and recruitment domain (CARD) and C-terminal domain [79–81]. A recent study showed that NS1 appears to decrease the ubiquitination of the CARD domains of RIG-I with increasing levels of NS1 [82]. Coimmunoprecipitation assays revealed that NS1 precipitated with the SPRY domain of TRIM25, an E3-ubiquitin ligase that conjugates K63 ubiquitin chains on several lysine residues in RIG-I CARDs including Lys172 that is important for RIG-I activation [83–85]. NS1 can also colocalize with MAVS, which is mitochondrial associated, in A549 cells infected with hRSV A2 [82,86]. NS1 also coimmunoprecipitates MAVS, suggesting that NS1 May be interfering with RIG-I signaling by binding to MAVS to further inhibit PRR activity [82]. NS1 localization at the mitochondria may be enhanced by the presence of NS2 and the formation of a heteromer [67]. The presence of NS1 on the mitochondria is consistent with a proteomics study that identified several mitochondrial proteins associated with NS1 [87]. NS1 may further suppress IFN signaling by facilitating the degradation of molecules in this pathway, including RIG-I, IRF3, and IRF7 [88] as well as TRAF3 and IKK?, although there are contrasting reports on this [88–90]. NS1 association with MAVS on the mitochondria along with NS2 can potentially serve as the formation site of a viral “degradasome” containing many IFN signaling proteins [88].

Suppression of RIG-I signaling by NS1 likely contributes to the decreased activation of several molecules downstream of RIG-I, including adapter protein TRAF3 and kinase IKK? that are important for the activation and phosphorylation of the transcription factor IRF3 [73,90]. IRF3 appears to be targeted independently by NS1 as transfection of NS1 coimmunoprecipitates IRF3 and interferes with IRF3 binding to its coactivator CBP and therefore prevents IRF3 translocation into the nucleus and activation of the IFNβ promoter [69,89].

NS1 not only impacts IFN production but also IFN responses. RSV infection inhibits signaling through the JAK/STAT pathway and specifically targets STAT2 [91]. NS1 has been shown to decrease STAT2 levels by facilitating its proteasomal degradation. Addition of protease inhibitors, mainly MG132, partially reduced STAT2 degradation [89,90,92–94]. NS1 also may be mediating degradation of STAT2 by forming an E3 ligase complex along with Cul2 and elongin C that targets STAT2 for K48-linked polyubiquitination and its subsequent degradation [92,95]. In addition, hRSV NS1 can promote proteasome-dependent degradation of 2’-5’ oligoadenylate synthase-like protein (OASL), which is an IFN-inducible antiviral protein, further suggesting that there are multiple steps or cellular antiviral proteins that NS1 inhibits to evade cellular antiviral innate immune responses [96].

## Nuclear NS1-mediated inhibition of the IFN signaling pathway

Although NS1 targets the function of many cytosolic host proteins, several subsequent studies suggested that NS1 may have a regulatory role in the nucleus even though hRSV replicates in the cytoplasm. NS1 was shown to partially localize in the nucleus of transfected 293T and A549 cells [67,69,87,90,97–100]. NS1 was shown to coimmunoprecipitate with histone H2B and stimulate its ubiquitination, potentially through an E3 ligase containing Elongin C and Cul2 [92,99], and results in HOX B5 and B6 gene expression in human bronchial epithelial cells. A subsequent proteomics study used EGFP-tagged NS1 to selectively precipitate interacting proteins in 293T cells and identified several nuclear associated proteins [87]. Among these were components involved in transcriptional regulation, including cyclin C, RNA polymerase II, and several subunits of Mediator complex, which are part of the preinitiation complex required for RNA polymerase II transcription. This was also verified in other independent studies, including one using StreptII-HA-tagged NS1 that identified 13 other protein subunits of Mediator complex, including Med1, Med14, and Med25, as an interactor of NS1 [100], one using three different screens (BioID, MAPPIT, and KISS) [101], and a yeast two-hybrid screen [102].

Consistent among these studies, are findings indicating that NS1 partially localizes in the nucleus. However, it was not clear whether this occurs in the context of hRSV infection. A recent study demonstrated that NS1 is present in the cytoplasm and in the nucleus of primary human tracheobronchial epithelial cells (hTBECs) when infected with hRSV [100]. This was further supported in hRSV infected human monocytic-derived dendritic cells as well as in the A549 cell line [100]. Moreover, when airway epithelial cells were treated with KPT-335, an inhibitor of exportin XPO1-mediated cargo transport from the nucleus, NS1 accumulated in the nucleus, suggesting that NS1 is not diffusing through the nuclear pore [100]. NS1 was found to be chromatin associated, and chromatin immunoprecipitation revealed that NS1 binds to regulatory elements (specifically promoters and enhancers) along with Mediator complex and suppresses the transcription of interferon stimulated genes, including IFIT2, IFIT3, and OAS1 [100]. This finding is consistent with earlier observations that suppression of antiviral gene expression is mediated by NS1 [96,97,103,104]. For example, NS1 may potentially inhibit JAK/STAT signaling by upregulating the gene expression of the suppressor of cytokine signaling proteins that are negative feedback inhibitors of JAK activity [103]. Transfection of A549 cells with NS1, and in combination with NS2, appears to increase the suppressor of cytokine signaling 1 and 3 (SOCS1 and SOCS3) expression, leading to lower levels of antiviral 2,5-OAS1 and MxA expression [105–107]. Furthermore, NS1 appears to interfere with SOD2 expression in response to hRSV infection [103]. SOD2 is a mitochondrial protein that is upregulated in response to oxidative stress stimulated by viral infection to promote cell survival and viral pathogenesis. While NS1 does not appear to directly bind to SOD2, infection of A549 cells with a recombinant hRSV virus lacking NS1 results in higher expression of SOD2 compared to mock infected cells, suggesting that NS1 modulates SOD2 expression.

## Structural basis for NS1 function

Biochemical and biophysical characterization of NS1 have started to elucidate the structural basis for the many roles of NS1. Several studies revealed that NS1 behaves as a monomer in solution [63,78,97,108]. However, other studies noted a potential for NS1 to form dimers [78]. The X-ray crystal structure of recombinant NS1 showed that NS1 is a monomer comprised of a beta sandwich flanked by three alpha helices (Figure 1) [97]. Notably, the third alpha helix appears to be extended away from the core of the protein, suggesting some conformational flexibility and perhaps explaining its potential to oligomerize and aggregate [109]. However, this alpha helix also contains several hydrophobic residues that are solvent accessible in the crystal structure and that is more likely to be involved in protein–protein interactions. Atomistic computer simulations also suggest that this C-terminal helix is more stable in the context of the NS1 structure than the isolated helix alone [97]. The four terminal Asp-Leu-Asn-Pro residues in NS1 that are common to both NS1 and NS2 are unstructured. A search for structural homologs revealed that NS1 adopts a similar structural fold to the N-terminal domain of the RSV matrix protein, with a backbone RMSD of 4.15 Å over 88 residues, despite having low sequence similarity [97]. NS1 contains an additional strand in the beta sandwich as well as an additional alpha helix at its C-terminus that is lacking in either subdomain of the hRSV matrix (M) protein [110]. Interestingly, prior studies of M protein showed that M partially localizes to the nucleus early during hRSV infection [34,111–113] like many matrix proteins from other negative strand RNA viruses [114–116]. Given the similarity in structure to hRSV M, it is not clear if the NS1 core is important for mediating entry into the nucleus since NS1 is lacking a canonical nuclear localization sequence. Mutation in the C-terminal helix does not affect nuclear localization of NS1 [100].

However, mutational studies on the NS1 C-terminal helix did result in a loss of IFNβ inhibition in vitro as well as a loss of suppression of dendritic cell maturation [97], suggesting that this unique helix is critical for NS1-mediated IFN antagonism and modulation of immune responses. Recombinant viruses containing NS1 mutations in the C-terminal helix displayed attenuated viral replication rates in A549 cells and resulted in significant changes in gene expression when compared to WT hRSV infection [97,117,118]. RNA-seq analysis of A549 cells transfected with the NS1 C-terminal helix Y125A mutant revealed significantly lower levels of gene expression of several antiviral genes, including IFIT2, IFIT3, IRF2, ISG20, and OAS1 [100]. While ChIP-qPCR showed that the NS1 Y125A mutant bound to Mediator complex at levels comparable to NS1 wildtype (WT), NS1 Y125A also displayed reduced capacity to inhibit ISRE reporter activity in vitro [100]. Collectively, results from these studies suggest that NS1 likely displaces one or more transcription factors that interact with Mediator complex to regulate transcription of antiviral gene expression and that this is mediated through interactions with the NS1 C-terminal helix. Interestingly, NS1 may be similar to other viral factors that target Mediator to stimulate transcriptional activation of viral genes [119–121]. Additional studies support that the NS1 C-terminal helix binds directly to Med25 and prevents interactions between transcriptional activators and Mediator complex in vitro [100–102]. The mechanistic details of this interaction or with other Mediator subunits have yet to be determined. However, these findings do not also rule out the possibility that other regions in NS1 are important for facilitating additional interactions given its multifunctional nature.

## NS2-mediated inhibition of the IFN signaling pathway

Like NS1, NS2 is a small nonstructural protein that functions to suppress IFN signaling. Early studies showed that recombinant viruses where the NS2 gene is deleted (ΔNS2) are viable but have slower growth kinetics, suggesting that NS2 is not critical for hRSV replication [71]. Infection of cells with recombinant ΔNS2 deletion viruses resulted in increased IFNβ mRNA levels; however, the levels were not as high as compared to recombinant virus with a NS1 gene deletion or NS1/NS2 gene deletions [69–72]. Altogether, these results suggested that NS2 May be functionally redundant with NS1 and may not be as potent of an IFN antagonist compared to NS1.

Subsequent studies demonstrated that NS2 targets different molecules in the IFNβ signaling pathway than NS1. In airway epithelial cells, RSV infection induces the expression of the PRR RIG-I during the early phase of infection. siRNA knockdown of RIG-I inhibited the subsequent activation of transcription factors IRF3 and NF?B [122]. Expression of NS2 inhibits RIG-I mediated IRF3 activation when stimulated with a 5’ppp RNA [64]. Subsequent coimmunoprecipitation experiments revealed that NS2 can bind directly to RIG-I, likely through interactions with the N-terminal CARD domains [64,123]. While RIG-I appears to have a greater role in response to hRSV infection, more recent studies suggest that the other cytosolic RIG-I-like receptor MDA5 may also contribute to host responses. MDA5 expression appears to be upregulated in hRSV-infected infants [124] and MDA5 colocalizes with MAVS and hRSV genomic RNA [125]. Consistent with this, NS2 was also shown to bind to MDA5 through the N-terminal CARDs in coimmunoprecipitation and in vitro pulldown assays [123]. However, there is no binding to MAVS or the MAVS CARD, suggesting that NS2 prevents IFN signaling at the level of the PRRs [123]. Additional studies demonstrated that NS2 binding reduces the overall levels of ubiquitination of RIG-I and MDA5, indicating that NS2 binding prevents RLR activation and signaling (Figure 3) [123].

NS2 also targets molecules downstream of the PRRs. Expression of NS2 decreased STAT2 expression levels when transfected into human tracheobronchial epithelial cells [93,126]. Furthermore, RNAi of NS2 expression in cells infected with RSV resulted in a loss of STAT2 inhibition, supporting a role of NS2 in STAT2 degradation that may be mediated by the *N*- and C-termini of NS2 [67]. Moreover, a ΔNS2 deletion virus lost the ability to suppress expression of STAT2 and PKR, an antiviral gene, when compared to WT or a ΔNS1 deletion virus [93]. This suggests that STAT2 inhibition is uniquely attributed to NS2. Interestingly, introduction of select mutations into NS2 and recombinant hRSVs resulted in an overall decrease in cellular ubiquitination, including of STAT2 proteins. In addition, these NS2 mutations resulted in a recombinant hRSV that is attenuated with decreased replication and growth kinetics in A549 cells, indicating a potential correlation between NS2 expression, ubiquitination, and antagonism of innate immune signaling that contributes to viral replication [94]. NS2 may also contribute to the degradation of STAT2 as part of a larger complex along with NS1 [67,88] and with other host proteins, such as MAP1B, an adaptor and scaffold protein [67]. The molecular mechanisms through which NS2 mediates these functions require further investigation.

## Structural basis for NS2 function

Based upon sequence alignments of the hRSV nonstructural proteins, there is little sequence similarity between NS2 and NS1, which is consistent with NS2 displaying functions that are distinct from NS1. Early biochemical characterization of NS2 revealed that recombinantly expressed NS2 behaved primarily as a monomeric protein that was unstable with a short half-life [78]. However, a more recent study demonstrated that recombinantly expressed NS2 is well behaved and can be purified to homogeneity [123]. Recombinant NS2 exists as a monomer in solution when analysed by size-exclusion chromatography coupled to multi-angle light scattering. Using this material, the X-ray crystal structure of NS2 was solved to 2.8 Å resolution (Figure 1). This structure revealed that NS2 is comprised of a single globular domain with a mixed alpha/beta fold formed by four alpha helices followed by a three-stranded, antiparallel beta sheet [123]. Comparison of the NS2 structure to other available structures in the Protein Data Bank (PDB) did not yield any hits with significant structural homology, including hRSV NS1. The C-terminal Asp-Leu-Asn-Pro motif that is common between the two nonstructural proteins are missing electron density, suggesting that this region is disordered and/or dynamic. Further analysis using hydrogen-deuterium exchange coupled to mass-spectrometry (HDX-MS) revealed that much of the NS2 structure also appears to be conformationally dynamic [123]. High levels of deuterium uptake are observed for a significant portion of the molecule, particularly at the N-terminus and loops connecting secondary structural elements. The N-terminal region of NS2 becomes protected from deuterium exchange upon bind to the RIG-I CARD domains. These results were validated through mutational analysis of the NS2 N-terminal residues. NS2 mutants display a loss of binding to RIG-I and reduced suppression of IFNβ mRNA levels [123]. Thus, one mechanism by which NS2 inhibits IFN signaling is by binding the PRRs RIG-I and MDA5 and preventing their activation and sustained downstream signaling, effectively limiting host immune responses to hRSV infection.

## Additional roles of NS proteins in RSV infection

While activities related to IFN inhibition by the hRSV NS proteins have been well documented, the NS proteins are multifunctional and have additional roles beyond antagonism of innate immune responses. Deletion of the NS1 gene, either alone or in combination with the NS2 gene, was associated with reduced virus replication [68–72]. A subsequent study revealed that substitution of NS1 and NS2 with parainfluenza virus 5 V protein cannot replace the functions of NS1 and NS2 in viral replication even in IFN-deficient Vero cells, indicating that NS1 and/or NS2 are important for viral replication [127]. The importance of the NS proteins in facilitating viral replication may be due in part to a role in inhibiting apoptosis and activating pro-survival pathways such as PI3K/AKT. Knockdown of one or both RSV NS proteins by siRNA promoted early apoptosis and NS-dependent suppression of apoptosis was observed in Vero cells, indicating that such inhibition is not related to type I IFN production [128]. In addition, the NS proteins suppressed TNF-induced apoptosis, which enhanced RSV growth [128]. RSV NS1 may also limit apoptosis by suppressing miR-24 expression, thereby inducing the transcription factor KLF6 expression to promote TGF-β-mediated cell cycle arrest, which facilitates RSV replication [129].

The nonstructural proteins also have roles in modulating host adaptive immunity. Infection with ΔNS2 deletion or ΔNS1/2 deletion RSVs results in increased levels of hRSV-specific pulmonary CD8(+) cytotoxic T lymphocytes, indicating that the NS2 protein, and not NS1, suppresses viral replication and the cytotoxic T cell response through inhibition of type I IFN responses in a mouse model [68]. Furthermore, autophagy in dendritic cells (DC) may play a role in hRSV infection that is facilitated by NS2 through interactions with Beclin-1 [130]. In contrast, other studies suggest that NS1 protein inhibits dendritic cell (DC) maturation as ΔNS1 deletion or ΔNS1/2 deletion RSVs or mutation of NS1 protein results in increased expression levels of DC maturation markers and cytokines [97,131]. Decreased antigen presentation and T-lymphocyte activation may be related to the IFN antagonism mediated by the NS1 protein [131]. However, subsequent studies demonstrated that NS1 protein suppresses activation and proliferation of CD103^+^ CD8^+^ T cells and affects the polarization of CD4^+^ T cells by co-cultivation of human DC and T cells. These effects were found to be associated with reduced maturation of dendritic cells caused by the NS1 protein and not due to suppressed IFN production, suggesting that the NS proteins have effects on the adaptive immune response independent on their IFN antagonist activities [132].

Interestingly, NS2 has also been implicated in promoting epithelial cell shedding of RSV-infected ciliated cells in the airway epithelium, perhaps through interactions with the cytoskeleton, leading to distal airway obstruction [2]. These results suggest that NS2 may play a role in promoting airway obstruction and therefore enhanced spread of infection, although the precise mechanisms by which NS2 protein mediates this remain to be defined [2].

## Vaccines

Early studies in the 1960s using a vaccine containing formalin-inactivated RSV failed to protect young children against natural exposure to hRSV infection and resulted in enhanced disease [133–135], which hampered the development of vaccines to hRSV. Subsequent attempts focused on developing live-attenuated vaccines, including cold-passaging or chemical mutagenesis, but were found to have varying levels of attenuation in naïve or exposed children and adults making this method of vaccine development unreliable [136]. Additional attempts with the advent of reverse genetics for hRSV [137–139] included gene deletions of NS1 and/or NS2 (and other genes such as M2–2) to elicit increased IFN responses by removal of their IFN antagonist activities. Not surprisingly, these deletion viruses resulted in over attenuated RSVs that failed to replicate sufficiently and elicit a durable response. Many advancements have been made since these initial attempts 60 years ago that have emerged from an increased understanding of the structure and function of F. As neutralizing antibodies target the prefusion conformation of F, this conformation has been the focus of several vaccine developments [13,14,140–142], including particle-based and maternal vaccines. The recent FDA-approved Arexvy uses a prefusion conformation of F that is stabilized by mutations as a particle-based vaccine for older adults; other vaccines in late-stage clinical trials also use a similar platform. While protection is now available for older adults and for for children under 6 months of age (through maternal vaccination), this still leaves a gap in protection for children between 6 months and 5 years of age. Because particle-based vaccines may lead to vaccine enhanced disease in young children, additional studies and development of other modalities, including RNA and live-attenuated vaccines, are needed. Recent advances in our understanding of multifunctional nature of NS1 and NS2 coupled with the availability of reverse genetics systems for RSV provides additional opportunities to develop new tools to enhance our understanding of hRSV infections and to better assess the efficacy of candidates. The X-ray crystal structures of NS1 and NS2 provide high-resolution insights into the molecular basis for viral-host interactions. Use of these detailed structural descriptions and defined immune evasion mechanisms presents an opportunity for rational approach to identifying critical interfaces or residues on NS1 and NS2 that are important for modulating host responses. This also provides an alternative method of fine-tuning attenuation by combining mutations in RSV vaccine candidates while maintaining a desired level of immunogenicity and that can be tested in different infection models. Thus, having multiple strategies will provide many advantages towards developing protection and reducing the global morbidity and mortality burden of hRSV infections.

## Data Availability

Data sharing is not applicable to this article as no new data were created or analysed in this study
